# Impact of non‐genetic heterogeneity of BRAF‐mutant colon cancer organoids on growth kinetics, drug sensitivity and Wnt dynamics

**DOI:** 10.1002/ijc.70453

**Published:** 2026-03-15

**Authors:** Viktoria Zieger, Ellen Woehr, Jasmin Traichel, Tilman Brummer, Roland Zengerle, Sabrina Kartmann, Stefan Zimmermann

**Affiliations:** ^1^ Laboratory for MEMS Applications, IMTEK ‐ Department of Microsystems Engineering University of Freiburg Freiburg Germany; ^2^ Hahn‐Schickard Freiburg Germany; ^3^ Study Program Molecular and Technical Medicine, Faculty Medical and Life Science University of Furtwangen Villingen‐Schwenningen Germany; ^4^ Institute of Molecular Medicine and Cell Research (IMMZ), Faculty of Medicine University of Freiburg Freiburg Germany; ^5^ Faculty of Biology University of Freiburg Freiburg Germany; ^6^ German Cancer Consortium (DKTK) Partner Site Freiburg and German Cancer Research Center (DKFZ) Heidelberg Germany; ^7^ Centre for Biological Signaling Studies BIOSS University of Freiburg Freiburg Germany; ^8^ Freeze‐O Organoid Bank, University Medical Center, Faculty of Medicine University of Freiburg Freiburg Germany

**Keywords:** 3D cell culture, automation, colon organoids, functional screening, organoid heterogeneity, Wnt pathway

## Abstract

Patient‐derived organoid (PDO) models are powerful systems for studying tumor biology and drug response. By retaining genetic, histological, and functional characteristics of the original tumor, including intra‐ and interpatient heterogeneity, they provide powerful tools to investigate therapy resistance. While genomic profiling is well established in organoid‐based screening, non‐genetic parameters such as organoid size, seeding density, and morphology remain underexplored, despite their potential to influence functional readouts. Here, we systematically examined how these factors affect proliferation, drug sensitivity, and Wnt responses in a murine colorectal cancer organoid model carrying oncogenic *Apc*, *Braf*, and *Trp53* mutations. Using our automated Pick‐Flow‐Drop handling platform, we implemented a reproducible plating workflow enabling precise control over organoid selection and screening conditions. We found that higher seeding density and larger organoid size reduced metabolic activity and decreased sensitivity to the MEK inhibitor trametinib. Moreover, distinct morphological subgroups, such as solid and cystic organoids, displayed differential drug responses under growth factor‐deprived conditions, correlating with distinct Wnt‐3a profiles in the culture supernatant. Solid organoids were more trametinib‐sensitive and exhibited higher Wnt‐3a levels, suggesting divergent cell compositions and pathway dependencies. Our findings highlight the functional relevance of non‐genetic variability in organoid cultures and establish a framework to improve reproducibility and biological insight in PDO‐based drug screening.

## Graphical abstract

What's new?

Patient‐derived organoids (PDOs) model tumor biology and drug response while preserving key tumor features, yet the impact of non‐genetic culture variables is poorly understood. This study examined the influence of organoid size, seeding density, and morphology on growth, drug sensitivity, and Wnt signaling in a colorectal cancer organoid model. Higher seeding density and larger organoids were linked to both decreased proliferation and sensitivity to the MEK inhibitor trametinib. In growth factor‐deprived conditions, solid organoids had higher trametinib sensitivity and higher Wnt‐3a levels than cystic forms. The presence of non‐genetic variability underscores the need for standardized organoid culture conditions.

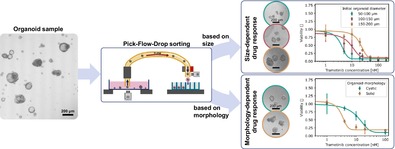

**Abbreviations**
3D: three‐dimensional
ATP: adenosine triphosphate
BME: basement membrane extracts
COCM: base medium (colon organoid culture medium)
COL: colorectal
CRC: colorectal cancer
CVAT: Computer Vision Annotation Tool
DMEM: Dulbecco's modified Eagle's medium
DMSO: dimethyl sulfoxide
ELISA: enzyme‐linked immunosorbent assay
GF: growth factor
IC_50_
: half maximal inhibitory concentration
MEKi: MEK inhibitor
MWP: microwell plate
n.s.: non‐significant
PDO: patient‐derived organoid
PFD: Pick‐Flow‐Drop
s.d.: standard deviation

## 1 INTRODUCTION

Tumor heterogeneity presents a significant challenge in drug development and therapy planning, often leading to treatment resistance and variable clinical outcomes.^1^, ^2^ While genomic profiling of tumor biopsies can identify driver mutations, it frequently fails to capture functional differences arising from non‐genetic factors such as epigenetic states, signaling dynamics, and microenvironmental influences.^3^ Patient‐derived organoids (PDOs) have emerged as powerful three‐dimensional (3D) models. By retaining the genetic, histological, and functional properties of the original tumor, including both intra‐ and interpatient variability, PDOs recapitulate tumor‐specific signaling activity, differentiation status, and drug responses, making them ideal for preclinical screening.^4^, ^5^, ^6^, ^7^, ^8^, ^9^

As shown by Kratz et al., 2025, even genetically identical organoid subclones can display divergent drug responses, highlighting the impact of non‐genetic heterogeneity.^10^ This often manifests in diverse organoid sizes, morphologies, and cellular compositions, which often correlate with functional states such as viability, metabolism, and pathway activation.^2^, ^11^, ^12^, ^13^, ^14^ Several studies have demonstrated that organoid morphology serves as a meaningful proxy for biological function, capable of predicting therapeutic responses and assisting in tumor classification.^13^, ^15^, ^16^ In addition to intrinsic heterogeneity, preparation‐induced variability, such as inconsistent organoid seeding density, can significantly impact functional results.^17^, ^18^, ^19^ Standardizing these baseline screening conditions is essential for improving reproducibility and interpretability in organoid‐based screenings.

Traditional screening approaches often fail to resolve such differences, as most assays rely on bulk organoid measurements that mask organoid‐to‐organoid variability.^14^, ^18^, ^20^, ^21^, ^22^ Current methods for heterogeneity‐resolved screening, such as fluorescence imaging,^23^ high‐content imaging coupled with deep learning that requires large, well‐annotated training datasets,^16^ or genetic profiling^10^ remain technically demanding, time‐consuming, and often overlook phenotypes determined by non‐genetic factors.

In this study, we employed our in‐house‐developed Pick‐Flow‐Drop (PFD) platform to enable automated, selective handling of organoids.^24^ The platform ensures high‐precision deposition and tight control over the plated organoid number, additionally allowing us to isolate specific subgroups from heterogeneous bulk samples based on optical features such as size or morphology. This enabled systematic investigation of how heterogeneity influences organoid functionality. To systematically evaluate preparation‐induced variability, we also intentionally varied organoid seeding density, which is a key source of variability in manual workflows. As a model, we used an intensively characterized murine colon organoid line harboring triple mutations in *Apc*, *Braf*, and *Trp53*, which closely mimics the transcriptomic landscape of a particularly aggressive subset of human BRAF‐mutant colorectal cancers (CRCs).^25^ Originally considered to occur only at low frequencies, CRCs with this triple mutant genotype have been detected at frequencies between 29% and 35%.^26^, ^27^, ^28^ Like their human counterparts, the phenotype of these organoids is driven by constitutive activation of the BRAF/MEK/ERK pathway, as the clinically relevant MEK inhibitor (MEKi) trametinib suppresses their growth. CRC is known for its high degree of genomic and non‐genetic heterogeneity, with substantial variability across molecular subgroups and between patients, which compromises therapy response.^29^, ^30^, ^31^ Understanding its underlying mechanisms can be facilitated by isolating organoids representing distinct subgroups for detailed functional analysis. Our results reveal how non‐genetic heterogeneity influences organoid growth, metabolic activity, sensitivity to MEK inhibition and Wnt‐3a signaling dynamics. These findings demonstrate the utility of automated, selective organoid handling for enabling reproducible, high‐resolution functional screening.

## 2 MATERIALS AND METHODS

### 2.1 Organoid culture

For this study, we used a previously established murine colorectal (COL) organoid model derived from mice harboring the *Braf* ^floxV600E/+^, *Trp53* ^LSL‐R172H/+^, *Apc* ^flox/flox^ and Villin::CreER^T2^ alleles, as described in Ref. [25]. Oncogene activation was induced in these so called BPAC organoids with 4‐hydroxytamoxifen (3 μM for 24 h). Organoids were cultured in either base medium (COCM) or growth factor‐enriched medium (COCM+GF). A detailed description of the media compositions is provided in Table 1. In brief, COCM consisted of Advanced DMEM/F12 supplemented with HEPES, L‐glutamine, B‐27, N‐2, N‐acetyl‐cysteine, and penicillin/streptomycin. COCM+GF was further supplemented with Noggin, R‐Spondin‐1, Wnt‐3a, EGF, CHIR‐99021, and Y27632. Although Wnt‐3a supplementation might no longer be required following 4‐hydroxytamoxifen induced CreER^T2^ mediated *Apc* deletion, we kept this factor in the medium to avoid additional variables. Indeed, as this ligand also signals via two APC‐independent, so‐called non‐canonical Wnt pathways,^32^ its absence might affect organoid growth even following the induction of oncogenic lesions.

**TABLE 1:** Media composition for organoid culture.

Media supplement	Supplier	Order number	Final supplement concentration/dilution factor	Base medium (COCM)	Growth factor‐enriched medium (COCM+GF)
Advanced DMEM/F12	Thermo Fisher Scientific (Gibco™)	12634028	na	+	+
HEPES	PAN Biotech	P05‐01100	10 mM	+	+
L‐Glutamin (200 mM)	Thermo Fisher Scientific (Gibco™)	25030081	2 mM	+	+
B‐27™ supplement (50×)	Thermo Fisher Scientific (Gibco™)	17504044	1:50	+	+
N‐2 supplement (100×)	Thermo Fisher Scientific (Gibco™)	17502048	1:100	+	+
N‐acetyl‐cysteine	Sigma‐Aldrich	A9165	1 mL	+	+
Penicillin–Streptomycin	Thermo Fisher Scientific (LifeTechnologies)	15140‐122	100 U/mL/100 μg/mL	+	+
Recombinant Murine Noggin	Thermo Fisher Scientific (PeproTech)	250‐38‐5UG	100 ng/mL	−	+
R‐Spondin‐1	Thermo Fisher Scientific (PeproTech)	194120‐38‐20UG	1 μg/mL	−	+
Recombinant Murine Wnt‐3a	Thermo Fisher Scientific (PeproTech)	315‐20‐10UG	50 ng/mL	−	+
mEGF	Thermo Fisher Scientific (PeproTech)	315‐09	50 ng/mL	−	+
CHIR‐99021 (CT99021)	Biomol GmbH, Germany (BPS Bioscience)	BPS‐27614‐2	3 μM	−	+
Y‐27632 dihydrochloride	Bio‐Techne GmbH (Tocris Bioscience)	1254	10 μm	−	+

Organoids were embedded in 4 mg/mL Cultrex BME (Reduced Growth Factor, Type 2, R&D Systems, Inc., USA; cat‐no. 3533‐005‐02) and seeded as 30 μL domes in 24‐well plates. For COCM+GF cultures, growth factors were also added to the matrix. After polymerization at 37°C for 30 min, 500 μL of medium was added. Media were replaced twice weekly with freshly added growth factors. Organoids were passaged weekly by mechanical and enzymatic dissociation using Accutase (Sigma‐Aldrich, USA; cat. no. A6964), followed by centrifugation and reseeding in fresh BME.

For functional assays, organoids were harvested 3 days post‐passaging by treating wells with 300 μL Cultrex Organoid Harvesting Solution (R&D Systems, USA) at 4°C for 30 min. Organoids were then collected by centrifugation, the matrix was removed, and organoids were resuspended in 4–5 mL COCM for downstream applications.

### 2.2 Automated organoid handling

For selective organoid handling prior to functional testing, we employed our in‐house fully automated Pick‐Flow‐Drop (PFD) platform.^24^ The system identifies and aspirates individual organoids from the harvested bulk organoid sample in a Petri dish based on predefined optical characteristics (pick), transports them through a controlled microfluidic flow within a capillary (flow), and precisely deposits them as nanoliter droplets into designated wells of a Microwell plate (MWP) using a non‐contact dispenser (drop). This gentle method preserves organoid viability (>95%) and structure, enabling selection by size or morphology and precise, reproducible plating of defined organoid numbers per well.^24^

Using the PFD platform, COL organoids were automatically plated into wells preloaded with 10 μL of 4 mg/mL BME matrix (supplemented with growth factors as needed) of a 384‐MWP (Multiwell Plate μClear white, Tissue‐culture treated surface; Greiner Bio‐One GmbH, Germany). The plate holder was cooled to 4°C to prevent premature matrix polymerization, ensuring dispensed organoids were fully embedded. After plating, MWPs were incubated at 37°C for 30 min to polymerize the matrix, followed by addition of 80 μL culture medium per well.

### 2.3 Image analysis and quantification of organoid growth

Organoid sizes were measured using an inverted light microscope at 4× magnification (CKX41, Olympus K.K., Japan). Images were preprocessed with the Computer Vision Annotation Tool (CVAT.ai) for aggregate labeling. The area A of segmented organoids was quantified using custom Python scripts based on the scikit‐image library.^33^ The organoid growth rate was calculated as:

$$\text{Growth rate}=\frac{A_{8}-A_{1}}{A_{1}}$$

where $A_{1}$ is the total organoid area per well on day 1 post plating and $A_{8}$ is the total organoid area per well on day 8, the final day of the functional screening.

### 2.4 Drug treatment

Trametinib (GSK1120212/JTP‐74057; Selleck Chemicals, Houston, TX, USA) was selected as an active compound as it is a clinically approved MEKi used to treat various solid tumor entities with BRAF^V600E^ mutations, usually in combination with dabrafenib.^34^ Trametinib has been trialed as part of drug combinations in colorectal cancer, although the results from the BEACON trial have now shifted the attention to another MEKi, binimetinib, which is applied in combination with encorafenib and cetuximab.^35^ Nevertheless, we chose trametinib as this allosteric MEKi is highly specific and efficient in suppressing the growth of our colon organoid model as a single substance,^25^ allowing us to assess proliferation and drug sensitivity while resolving effects of non‐genetic variation. The trametinib concentration range was chosen based on our previous work with this organoid model^25^ and plasma levels observed in patients.^36^ Working concentrations were prepared by diluting the stock solution in DMSO, which was also used in negative controls at equivalent concentrations. Organoids were treated on day 1 after plating by replacing the medium with fresh COCM or COCM+GF containing the desired trametinib concentration. Treatments were repeated on days 4 and 7, and cell viability was assessed on day 8.

### 2.5 Viability analysis

Viability was determined using the CellTiter‐Glo 3D Viability Assay (Promega Corporation, USA), which quantifies ATP as an indicator of metabolically active cells through luminescence. Prior to organoid measurements, assay performance was verified by generating an ATP standard curve according to the manufacturer's instructions, confirming a linear relationship between ATP concentration and luminescence. Luminescence was measured with a Tecan Spark20M plate reader and background‐corrected using signals from cell‐free wells. Viability was normalized to the mean signal of DMSO controls and adjusted by the number of organoids per well.

### 2.6 Wnt‐3a analysis

To assess Wnt‐3a levels in the supernatant of COL organoids cultured in COCM without additional growth factors, medium was collected during exchanges on days 1, 4, and 7 and stored at −80°C. Wnt‐3a concentrations were measured using an enzyme‐linked immunosorbent assay (ELISA; Mouse Wnt‐3a DuoSet ELISA and Ancillary Reagent Kit, cat. no. DY1324B‐05 and DY008B, Bio‐Techne GmbH, Germany) according to the manufacturer's instructions. Measurements were performed in technical duplicates per well, and mean values were calculated.

### 2.7 Statistical analysis

The number of replicates *n* are indicated in the respective figure legends. Box plots display numerical data by illustrating quartiles, with the box spanning from the first quartile (Q1) to the third quartile (Q3), representing the middle 50% of the data. Whiskers extend to the most extreme data points within 1.5 times the interquartile range. The box's center line indicates the median. Error bars represent ± standard deviation (s.d.) around the mean. Statistical analyses were conducted using Python's SciPy module (scipy.stats).^37^ Data normality was assessed via the Shapiro–Wilk test. Normally distributed data were analyzed with two‐tailed *t*‐tests, while non‐normal data or data where *n* < 8 were evaluated using the Mann–Whitney *U* test. *p* values ≥.05 were considered non‐significant (n.s.), with significance levels denoted as *p < .05, **p < .01, and ***p < .001.

Further information on the Organoid growth data (Table S1), CellTiter‐Glo® Cell Viability data (Table S2) incl. ATP standard curve data (Table S3), and Wnt3a ELISA assay data (Table S4) can be found in Tables S1–S4.

## 3 RESULTS

### 3.1 Seeding density affects organoid growth, metabolism, and trametinib sensitivity

**FIGURE 1:**
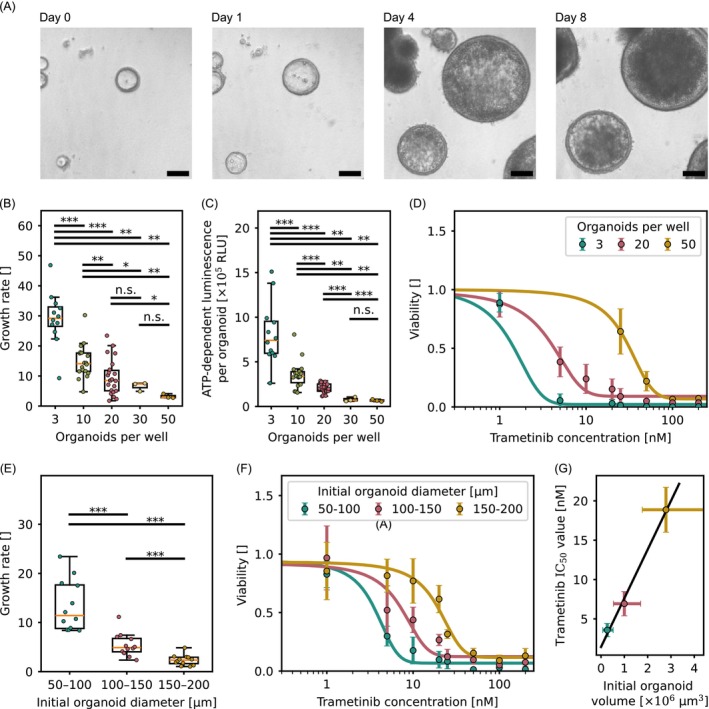
(A) Representative image of untreated COL organoids cultured in COCM+GF (Noggin, R‐Spondin‐1, Wnt‐3a, EGF, CHIR‐99021, Y27632) over 8 days. Scale bar: 100 μm. (B) Organoid growth rates at different organoid seeding densities (*n* = 12,18,22,3,3; statistical analysis: Mann–Whitney test). (C) Metabolic activity of untreated organoids on day 8, measured by CellTiter‐Glo 3D assay, normalized to the number of organoids per well (*n* = 12,18,22,3,3; statistical analysis: Mann–Whitney test). (D) Dose response to trametinib at different organoid seeding densities measured on day 8 post plating. Each condition: *n* ≥ 3. IC_50_ values were: Seeding density 3: 1.2 nM; Seeding density 20: 3.1 nM; Seeding density 50: 27.9 nM. (E) Organoid growth rates at varying initial organoid diameters (*n* = 12 per condition with 20 organoids per replicate; statistical analysis: unpaired two‐tailed *t*‐test). (F) Dose response to trametinib based on initial organoid diameters assessed on day 8 post plating (*n* = 6 per condition with 20 organoids per replicate). IC_50_ values were: 50–100 μm: 3.6 nM; 100–150 μm: 6.9 nM; 150–200 μm: 18.9 nM. (G) Correlation between initial organoid volume and derived IC_50_ values from (F), with a goodness‐of‐fit of *R* ^2^ = 0.994. Error bars on the *x*‐axis represent the range of initial organoid volumes; error bars on the *y*‐axis indicate uncertainty in IC_50_ values obtained from dose response curves.

Triple‐mutant COL organoids *Apc* ^∆/∆^, *Trp53* ^R172H/+^, and *Braf* ^V600E/+^ cultured in growth factor‐enriched medium (COCM+GF, Table 1) exhibited a characteristic cystic morphology. Organoids plated with the automated PFD platform showed robust growth while retaining their cystic architecture (Figure 1A). This is consistent with previous findings that the plating process preserves organoid viability.^24^

A major source of variability in organoid screening assays is inconsistency in seeding density, which leads to fluctuations in the number of organoids and cells per well. This variability often arises from manual pipetting, where precise control over the number of plated organoids is difficult to achieve. To investigate the impact of seeding density, we used the PFD platform to precisely plate COL organoids (50–120 μm in diameter) in defined numbers ranging from 3 to 50 per well and monitored their growth over 8 days, a time frame typical for organoid drug screening assays.^25^ Although all organoids, regardless of seeding density, exhibited substantial size increases over time (Figure S1A), higher seeding densities significantly suppressed growth rates, indicating density‐dependent growth inhibition (Figure 1B). Interestingly, ATP levels measured on day 8 using the CellTiter‐Glo 3D assay were comparable across all seeding densities (Figure S1B). Values shown in Figure S1B represent total ATP per well and therefore reflect the combined contribution of all organoids present within the well. These values suggest a density‐dependent metabolic adaptation in which total ATP production per well reaches a plateau, likely due to shared nutrient limitations and accumulation of metabolic byproducts within the well. Accordingly, when normalized to the number of organoids per well, ATP production per organoid decreased markedly with increasing seeding density (Figure 1C), indicating reduced metabolic activity under high culture density conditions. Treatment with the MEKi trametinib further revealed a strong seeding density‐dependent shift in drug sensitivity (Figure 1D). We did not aim to assess MEK inhibitor efficacy per se; rather, these experiments were designed to evaluate how seeding density modulates sensitivity to trametinib in terms of viability. COL organoids seeded at low density (3 per well) exhibited high sensitivity with an IC_50_ of 1.2 ± 1.0 nM, while 20 organoids per well and 50 organoids per well showed progressively reduced responses (20 organoids: 3.1 ± 1.5 nM; 50 organoids: 27.9 ± 7.0 nM). The same trend was observed in organoid cultures under growth factor‐depleted conditions (Figure S2), highlighting how initial seeding density affects functional assay results.

### 3.2 Organoid size determines growth rate and trametinib sensitivity

A second source of variability in organoid screening is size. This can result from intrinsic variation in growth rates within the sample, for example due to the original cellular composition, leading to a mixture of slow‐ and fast‐growing organoids. It can also arise from external factors, such as variations in passaging timing or differences in cultivation protocols.

To address this, we investigated how initial organoid size influences growth and drug sensitivity. Our COL organoid model cultured in COCM+GF displayed intrinsic size heterogeneity 3 days after passaging. Using the PFD platform, organoids within defined diameter ranges were selectively withdrawn from the size‐heterogeneous bulk sample and precisely deposited into wells according to their diameter. To isolate size as the variable, the seeding density was held constant at 20 organoids per well.

We observed a strong inverse correlation between initial size and growth rate (Figure 1E): smaller organoids (50–100 μm) grow faster than medium‐sized ones (100–150 μm), which in turn grow faster than large ones (150–200 μm). By day 8 post plating, however, the total organoid area per well converged across all size groups (Figure S1C). ATP‐dependent luminescence also showed no significant differences (Figure S1D), suggesting that organoids reached a growth and metabolic equilibrium, likely limited by nutrient availability and diffusion constraints within the organoid.

Further, also the trametinib response was strongly size‐dependent. As above, these experiments focus on heterogeneity‐driven modulation of drug response rather than demonstrating intrinsic MEK inhibitor activity. Organoids with initial diameters of 50–100 μm displayed an IC_50_ of 3.6 ± 0.8 nM, which increased to 6.9 ± 1.5 nM for 100–150 μm organoids and to 18.9 ± 2.9 nM for those in the 150–200 μm range (Figure 1F). Since the triple‐mutant COL organoids cultured in growth factor‐enriched conditions exhibited a highly spherical morphology, the average organoid diameter d per well on day 0 was used to calculate the mean organoid volume $V=\frac{4}{3}\pi {\left(\frac{d}{2}\right)}^{3}$ for further analysis of this relationship. Plotting the initial organoid volume against IC_50_ values revealed a linear correlation (Figure 1G), underscoring the influence of organoid size on drug sensitivity.

### 3.3 Cystic and solid organoids respond differently to trametinib in GF‐deprived conditions

As previously reported,^25^ the COL organoid model with triple mutation in *Apc*, *Trp53*, and *Braf* also grows under growth factor‐depleted conditions. When cultured in COCM supplemented with Noggin, R‐Spondin‐1, Wnt‐3a, EGF, CHIR‐99021, and Y27632 (COCM+GF), COL organoids maintained a highly cystic morphology (Figure S3A). In contrast, under growth factor‐deprived conditions (COCM alone), the organoids exhibited greater morphological intersample heterogeneity (Figure S3B). Three days post‐propagation, two distinct subgroups emerged: one with a predominantly cystic morphology and another with a more solid appearance. This raised the question of whether these morphological phenotypes reflect underlying functional differences.

To address this, we utilized the PFD platform for selection. An experienced researcher identified organoids in the reservoir that were associated with either subgroup, and the platform was used to automatically aspirate and deposit the selected organoids at a seeding density of 20 organoids per well. A size window of 50–120 μm was applied to minimize size‐dependent effects. We verified that plated solid and cystic organoids were within a comparable size range, as the diameters of the morphologically distinct subgroups did not differ significantly (Figure S4).

**FIGURE 2:**
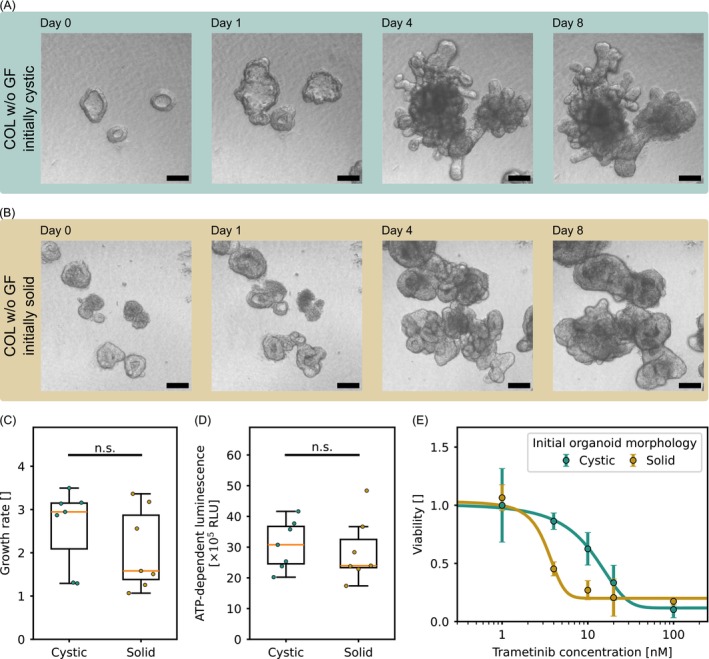
(A), (B) Proliferation and morphological plasticity of untreated COL organoids cultured in COCM without additional growth factors over an observation period of 8 days. Scale bar: 100 μm. (C) Organoid growth rate stratified by different organoid morphologies (*n* = 7 per condition with 20 organoids per replicate; statistical analysis: Mann–Whitney test). (D) Metabolic activity of untreated organoids on day 8, measured using the CellTiter‐Glo 3D assay, which quantifies ATP as a marker of viability (*n* = 7 per condition with 20 organoids per replicate; statistical analysis: Mann–Whitney test). (E) Drug response to increasing concentrations of trametinib in organoids with distinct morphologies, assessed by CellTiter‐Glo 3D assay on day 8 (*n* ≥ 3 per condition with 20 organoids per replicate). The derived IC_50_ values were: Cystic organoids: 8.8 ± 2.1 nM; Solid organoids: 3.2 ± 1.7 nM.

Imaging analysis over 8 days revealed notable morphological plasticity in both subgroups. In contrast to the stable cystic phenotype maintained in COCM+GF (Figure 1A), cystic organoids cultured in COCM without additional growth factors developed complex, branched structures with crypt‐like features (Figure 2A). Solid organoids cultured in COCM without additional growth factors maintained a compact architecture but began forming bud‐like protrusions, though with lower structural complexity (Figure 2B).

Despite these morphological differences, growth rates and metabolic activity as measured by ATP‐dependent luminescence were not significantly different between the two groups (Figure 2C,D). However, both parameters were substantially reduced compared to organoids cultured in COCM+GF as expected.

In contrast to their similar growth and metabolic profiles, the two subgroups exhibited considerable differences in trametinib sensitivity (Figure 2E). Initially, cystic organoids were less responsive, with an IC_50_ of 8.8 ± 2.1 nM, while solid organoids showed significantly greater sensitivity, with an IC_50_ of 3.2 ± 1.7 nM.

### 3.4 Differential Wnt‐3a dynamics linked to organoid morphology and seeding density under GF‐deprived conditions

**FIGURE 3:**
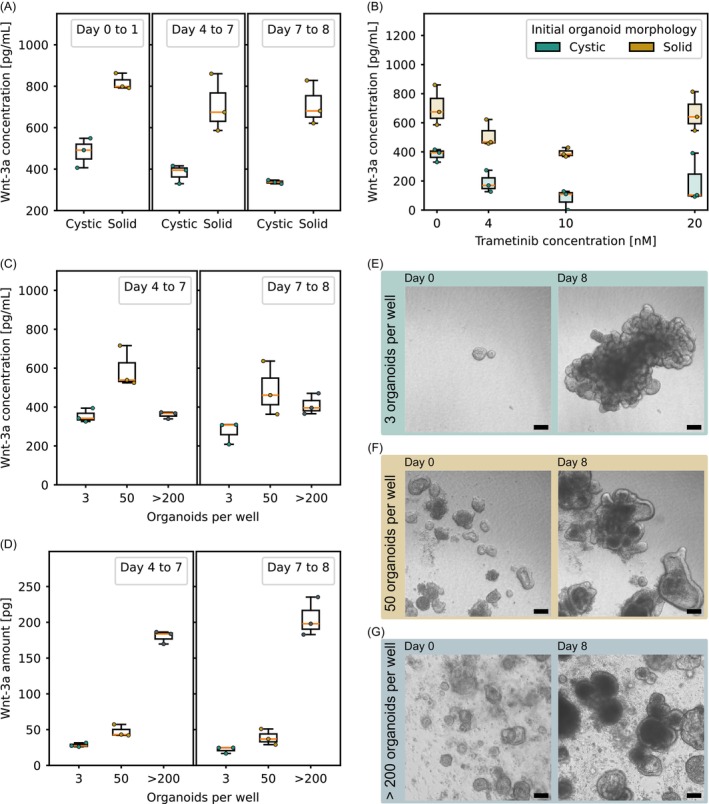
(A) Wnt‐3a concentration in the culture supernatant measured by ELISA in COL organoids cultured without additional growth factors. Supernatant was collected at medium exchange time points: Day 1 (24 h accumulation period), day 4 (72 h accumulation period), and day 7 (24 h accumulation period) (*n* = 3 per condition with 20 organoids per replicate). (B) Wnt‐3a concentration in supernatant measured by ELISA in response to varying trametinib concentrations (*n* = 3 per condition with 20 organoids per replicate). (C) Wnt‐3a concentration and (D) absolute Wnt‐3a levels in the culture supernatant measured by ELISA in COL organoids cultured without additional growth factors at varying organoid seeding densities. Supernatant was collected on day 4 (72 h accumulation period) and day 7 (24 h accumulation period). Seeding densities 3 and 50 were kept in 384‐well plate format with organoids growing within a 19 μL BME bed covered by 80 μL medium. Seeding density >200 was kept in 24‐well plate format with organoids growing in a 30 μL BME dome covered by 500 μL medium (*n* = 3 per condition). (E), (F), (G) Proliferation and morphological reorganization of untreated COL organoids over 8 days of culture without additional growth factors, analyzed in relation to different seeding densities. Scale bar: 100 μm.

To further explore the basis of the differential trametinib sensitivity and potential crosstalk of MAPK/ERK and WNT pathways,^38^, ^39^ we quantified Wnt‐3a levels in culture supernatants collected on days 1, 4, and 7 during medium and drug exchanges. Measured concentrations were consistent with previously reported values for organoid cultures lacking exogenous Wnt supplementation.^40^ Notably, supernatants from initially cystic organoids consistently exhibited lower Wnt‐3a levels than those from initially solid organoids throughout the 8‐day period (Figure 3A). The absence of increasing Wnt‐3a levels over time suggests a stable secretion‐uptake equilibrium that differs between the two morphological subgroups. Interestingly, in trametinib‐treated wells, Wnt‐3a levels initially decreased with increasing drug concentration but subsequently returned to near baseline levels, particularly in solid organoids (Figure 3B).

We also investigated how organoid seeding density affects Wnt‐3a levels under growth factor‐deprived conditions. Supernatants were collected from 384‐well plates seeded with either 3 or 50 mixed‐morphology organoids in a BME bed, and from base cultures in 24‐well plates containing over 200 organoids per well in a BME dome. While Wnt‐3a concentrations did not consistently increase with organoid number (Figure 3C), the total amount of Wnt‐3a measured in the supernatant considerably increased with increasing seeding densities (Figure 3D).

Under GF‐deprived conditions, organoid seeding density also affected morphology over the 8‐day period. While organoids initially appeared similar across conditions, morphological differences became apparent over time. In low‐density wells, organoids developed extensive budding (Figure 3E). This phenotype diminished with increasing organoid numbers, with high‐density cultures exhibiting a predominantly cystic morphology (Figure 3F,G), resembling that of organoids maintained in COCM+GF.

## 4 DISCUSSION

This study systematically explores how non‐genetic variables, such as organoid seeding density, size, and morphology, affect functional readouts in colorectal cancer organoid assays. Using our automated Pick‐Flow‐Drop platform, we achieved precise and reproducible organoid handling, allowing controlled interrogation of these variables in a model carrying oncogenic *Apc*, *Braf*, and *Trp53* mutations. The results from mouse organoids may not fully translate to human systems, and further validation in human PDOs is needed to confirm the findings. However, this model closely reflects the molecular landscape of an aggressive subset of human BRAF^V600E^‐driven colorectal cancer^25^, ^41^ and offers translational insight into how non‐genetic factors shape drug response and signaling behavior in this genetically defined and clinically challenging CRC subtype. We envisage that our technology will also provide new insights into non‐genetic dependencies of other CRC subtypes, for example, driven by KRAS alterations.

We found that higher seeding density and larger organoid size were associated with reduced proliferation and decreased trametinib sensitivity. These effects likely arise from a combination of factors, including nutrient, mitogen, or oxygen limitations and diffusion constraints in cultures with high seeding densities or large organoids. Increased cellular count may also reflect greater cellular heterogeneity, including quiescent, hypoxic, or differentiated cells less responsive to MEK inhibition.^11^

Beyond that, trametinib specifically targets proliferative, metabolically active cells via MEK inhibition in the MAPK pathway.^42^, ^43^ Thus, reduced metabolic activity in cultures with either dense seeding or large organoids may contribute to decreased drug response. The observed trend aligns with reports that drug efficacy is attenuated in quiescent or slowly cycling cells, which may be enriched under growth‐limiting conditions.^44^, ^45^

We also observed that organoids with either solid or cystic morphologies differently responded to trametinib under growth factor‐deprived conditions. Solid organoids were more sensitive and exhibited higher Wnt‐3a levels in their supernatant. This is an interesting observation, as increased Wnt signaling has been originally associated with the induction of a cystic morphology, at least in oncogene‐naïve small intestinal organoids supplemented with exogenous Wnt3a.^11^ Whether these contrasting Wnt‐3a associated effects reflect the profound differences between small intestinal and colonic organoids^25^ or can be explained by distinct dosage effects conferred by exogenously versus endogenously produced Wnt3a represents an interesting subject for further studies. Moreover, the different Wnt3a levels found in the supernatants of solid and cystic organoids may reflect divergent cell compositions or variations in Wnt secretion and uptake. For example, cystic organoids may contain more Wnt‐responsive LGR5^+^ cells, which internalize extracellular Wnt and reduce residual concentrations.^11^, ^13^ Solid organoids, conversely, may contain more differentiated cell populations and exhibit lower stemness,^13^, ^46^ which could make them more dependent on ERK‐driven proliferation, thereby increasing their sensitivity to trametinib. This interpretation is supported by the emergence of crypt‐like remodeling structures in initially cystic organoids over time, which are potentially indicative of a stem cell niche. However, without direct marker analysis or pathway activity assays, this remains a hypothesis, but an interesting area of future research. This is supported by a very recent publication reporting the interplay between the ERK and WNT‐pathways in shaping intestinal epithelial plasticity in malignant transformation and responses to targeted therapy.^47^ The Pick‐Flow‐Drop platform could facilitate these analyses through the rapid, well‐documented and gentle isolation of morphologically distinct phenotypes.

Interestingly, Wnt‐3a levels in solid organoids responded dynamically to trametinib exposure. Whether these dynamics reflect a feedback activation of Wnt signaling following ERK remains unclear. Such interactions, however, are well documented in CRC^38^, ^39^ and underscore the importance of considering potential signaling crosstalk in drug screening models.

Finally, seeding density influenced not only proliferation but also Wnt‐3a concentrations and morphological appearance under growth factor‐deprived conditions. Denser cultures showed higher total Wnt‐3a levels and favored a cystic phenotype, whereas low‐density cultures promoted budding. This supports prior observations that seeding conditions modulate the paracrine signaling environment and structural plasticity of organoids.^48^ The discrepancy between Wnt‐3a concentration and total Wnt‐3a levels in the supernatant likely arises from differences in diffusion dynamics across culture formats, such as 24 well plates with BME domes versus 384‐MWPs with BME beds.^40^

Together, these findings emphasize the critical impact of non‐genetic variability on functional screening results. Factors such as density, size, and morphology can mask or amplify drug responses, underscoring the need for tighter control and standardization in organoid‐based drug testing. Automated systems like the PFD platform offer a practical solution to achieve this control, enabling more reliable and stratified profiling in patient‐derived organoid models. While our study focused on a genetically defined murine *Apc*/*Braf*/*Trp53* organoid model, the approach provides a proof of concept for systematically differentiating organoid features and their impact on functional outcomes. Although exact results may vary across genetic backgrounds and human PDOs, this strategy can be applied to human samples to uncover context‐specific signaling dependencies and therapeutic vulnerabilities, with potentially even more pronounced effects in the inherently more heterogeneous human organoid systems. By accounting for non‐genetic variation, this approach can help to reveal functional effects shaped by cellular plasticity rather than genetic alterations, supporting more precise and interpretable drug response profiling across diverse cancer types.

## AUTHOR CONTRIBUTIONS

**Viktoria Zieger:** Conceptualization; methodology; formal analysis; investigation; data curation; visualization; writing – original draft. **Ellen Woehr:** Conceptualization; methodology; data curation; investigation; writing – review and editing; formal analysis. **Jasmin Traichel:** Conceptualization; methodology; writing – review and editing. **Tilman Brummer:** Conceptualization; validation; writing – review and editing; supervision. **Roland Zengerle:** Supervision; resources; writing – review and editing. **Sabrina Kartmann:** Conceptualization; resources; writing – review and editing; supervision; project administration. **Stefan Zimmermann:** Conceptualization; validation; writing – review and editing; supervision; project administration.

## FUNDING INFORMATION

This work was supported by the German Federal Ministry of Research, Technology and Space, research grants ADAPT‐2 (03LW0335K/03LW0336, SK/SZ) and PIPER (03LWH0076, SK/SZ), German Cancer Consortium DKTK (FR01‐376) and intramural support (FREEZE‐O shared facility).

## CONFLICT OF INTEREST STATEMENT

V.Z. and S.K. have submitted the basic concept of the Pick‐Flow‐Drop principle for patent. T.B. reports honoraria from PierreFabre and the European Society of Medical Oncology outside of the submitted work. The other authors declare no conflict of interest.

## Supporting information


**FIGURE S1:** Organoid growth and metabolic activity in dependence of seeding.
**FIGURE S2:** Dose response to trametinib at different organoid seeding.
**FIGURE S3:** Organoids cultured under growth factor‐supplemented conditions.
**FIGURE S4:** Verification of size similarity between plated cystic and solid.

**TABLE S1:** Organoid growth data.
**TABLE S2:** CellTiter‐Glo® cell viability data.
**TABLE S3:** CellTiter‐Glo® ATP standard curve data.
**TABLE S4:** Wnt3a ELISA assay data.

## Data Availability

The data that support the findings of this study are available in the supplementary materials and from the corresponding author upon reasonable request.

## References

[1] Worldwide Cancer Research . Why is cancer so hard to cure? 2025 https://www.worldwidecancerresearch.org/information-and-impact/cancer-myths-and-questions/why-is-cancer-so-hard-to-cure/

[2] Skala MC , Deming DA , Kratz JD . Technologies to assess drug response and heterogeneity in patient‐derived cancer organoids. Annu Rev Biomed Eng. 2022;24:157‐177. 35259932 10.1146/annurev-bioeng-110220-123503 PMC9177801

[3] Lauinger M , Christen D , Klar RFU , et al. BRAFΔβ3‐αC in‐frame deletion mutants differ in their dimerization propensity, HSP90 dependence, and druggability. Sci Adv. 2023;9:eade7486. 37656784 10.1126/sciadv.ade7486 PMC11804575

[4] Hoefflin R , Geißler A‐L , Fritsch R , et al. Personalized clinical decision making through implementation of a molecular tumor board: a German single‐center experience. JCO Precis Oncol. 2018;2:1‐16. 10.1200/PO.18.00105 PMC7446498 32913998

[5] Liu L , Yu L , Li Z , Li W , Huang W . Patient‐derived organoid (PDO) platforms to facilitate clinical decision making. J Transl Med. 2021;19:40. 33478472 10.1186/s12967-020-02677-2 PMC7821720

[6] Kim S‐Y , van de Wetering M , Clevers H , Sanders K . The future of tumor organoids in precision therapy. Trends Cancer. 2025;11:665‐675. 40185656 10.1016/j.trecan.2025.03.005

[7] Seppälä TT , Zimmerman JW , Suri R , et al. Precision medicine in pancreatic cancer: patient‐derived organoid pharmacotyping is a predictive biomarker of clinical treatment response. Clin Cancer Res. 2022;28:3296‐3307. 35363262 10.1158/1078-0432.CCR-21-4165 PMC9357072

[8] de Witte CJ , Espejo Valle‐Inclan J , Hami N , et al. Patient‐derived ovarian cancer organoids mimic clinical response and exhibit heterogeneous inter‐ and Intrapatient drug responses. Cell Rep. 2020;31:107762. 32553164 10.1016/j.celrep.2020.107762

[9] Li L , Knutsdottir H , Hui K , et al. Human primary liver cancer organoids reveal intratumor and interpatient drug response heterogeneity. JCI Insight. 2019;4:4. 10.1172/jci.insight.121490 PMC6413833 30674722

[10] Kratz JD , Rehman S , Johnson KA , et al. Subclonal response heterogeneity to define cancer organoid therapeutic sensitivity. Sci Rep. 2025;15:12072. 40200028 10.1038/s41598-025-96204-2 PMC11978853

[11] Merenda A , Fenderico N , Maurice MM . Wnt signaling in 3D: recent advances in the applications of intestinal organoids. Trends Cell Biol. 2020;30:60‐73. 31718893 10.1016/j.tcb.2019.10.003

[12] Marklein RA , Lam J , Guvendiren M , Sung KE , Bauer SR . Functionally‐relevant morphological profiling: a tool to assess cellular heterogeneity. Trends Biotechnol. 2018;36:105‐118. 29126572 10.1016/j.tibtech.2017.10.007

[13] Betge J , Rindtorff N , Sauer J , et al. The drug‐induced phenotypic landscape of colorectal cancer organoids. Nat Commun. 2022;13:3135. 35668108 10.1038/s41467-022-30722-9 PMC9170716

[14] Zhang L , Wang L , Yang S , He K , Bao D , Xu M . Quantifying the drug response of patient‐derived organoid clusters by aggregated morphological indicators with multi‐parameters based on optical coherence. Biomed Opt Express. 2023;14:1703‐1717. 37078050 10.1364/BOE.486666 PMC10110317

[15] Lee MR , Kang S , Lee J , et al. Organoid morphology‐guided classification for oral cancer reveals prognosis. CR Med. 2025;6:102129. 10.1016/j.xcrm.2025.102129 PMC12147898 40359934

[16] Huang K , Li M , Li Q , Chen Z , Zhang Y , Gu Z . Image‐based profiling and deep learning reveal morphological heterogeneity of colorectal cancer organoids. Comput Biol Med. 2024;173:108322. 38554658 10.1016/j.compbiomed.2024.108322

[17] DeStefanis RA , Kratz JD , Olson AM , et al. Impact of baseline culture conditions of cancer organoids when determining therapeutic response and tumor heterogeneity. Sci Rep. 2022;12:5205. 35338174 10.1038/s41598-022-08937-z PMC8956720

[18] Deben C , La Cardenas De Hoz E , Rodrigues Fortes F , et al. Development and validation of the normalized organoid growth rate (NOGR) metric in brightfield imaging‐based assays. Commun Biol. 2024;7:1612. 39627437 10.1038/s42003-024-07329-5 PMC11615385

[19] Du Y , Li X , Niu Q , et al. Development of a miniaturized 3D organoid culture platform for ultra‐high‐throughput screening. J Mol Cell Biol. 2020;12:630‐643. 32678871 10.1093/jmcb/mjaa036 PMC7751183

[20] Landon‐Brace N , Li NT , McGuigan AP . Exploring new dimensions of tumor heterogeneity: the application of single cell analysis to organoid‐based 3D in vitro models. Adv Healthc Mater. 2023;12:e2300903. 37589373 10.1002/adhm.202300903 PMC11468421

[21] Gehling K , Parekh S , Schneider F , et al. RNA‐sequencing of single cholangiocyte‐derived organoids reveals high organoid‐to organoid variability. Life Sci Alliance. 2022;5:5. 10.26508/lsa.202101340 PMC9348635 35914813

[22] Hof L , Moreth T , Koch M , et al. Long‐term live imaging and multiscale analysis identify heterogeneity and core principles of epithelial organoid morphogenesis. BMC Biol. 2021;19:37. 33627108 10.1186/s12915-021-00958-w PMC7903752

[23] Sharick JT , Walsh CM , Sprackling CM , et al. Metabolic heterogeneity in patient tumor‐derived organoids by primary site and drug treatment. Front Oncol. 2020;10:553. 32500020 10.3389/fonc.2020.00553 PMC7242740

[24] Zieger V , Frejek D , Zimmermann S , et al. Towards automation in 3D cell culture: selective and gentle high‐throughput handling of spheroids and organoids via novel pick‐flow‐drop principle. Adv Healthc Mater. 2024;13:e2303350. 38265410 10.1002/adhm.202303350 PMC11468932

[25] Reischmann N , Andrieux G , Griffin R , Reinheckel T , Boerries M , Brummer T . BRAFV600E drives dedifferentiation in small intestinal and colonic organoids and cooperates with mutant p53 and Apc loss in transformation. Oncogene. 2020;39:6053‐6070. 32792685 10.1038/s41388-020-01414-9 PMC7498370

[26] Vogel A , Murugesan K , Kendre G , et al. Association of RNF43 genetic alterations with BRAFV600E and MSIhigh in colorectal cancer. JCO Precis Oncol. 2024;8:e2300411. 38394466 10.1200/PO.23.00411

[27] van Morris K , Parseghian CM , Bahrambeigi V , et al. Phase 1/2 trial of encorafenib, cetuximab, and nivolumab in microsatellite stable BRAFV600E metastatic colorectal cancer. Cancer Cell. 2025;43:2106‐2118.e3. 40882637 10.1016/j.ccell.2025.08.002 PMC12431688

[28] Elez E , Ros J , Fernández J , et al. RNF43 mutations predict response to anti‐BRAF/EGFR combinatory therapies in BRAFV600E metastatic colorectal cancer. Nat Med. 2022;28:2162‐2170. 36097219 10.1038/s41591-022-01976-z PMC9556333

[29] Sobral D , Martins M , Kaplan S , et al. Genetic and microenvironmental intra‐tumor heterogeneity impacts colorectal cancer evolution and metastatic development. Commun Biol. 2022;5:937. 36085309 10.1038/s42003-022-03884-x PMC9463147

[30] Blank A , Roberts DE , Dawson H , Zlobec I , Lugli A . Tumor heterogeneity in primary colorectal cancer and corresponding metastases. Does the apple fall far from the tree? Front Med. 2018;5:234. 10.3389/fmed.2018.00234 PMC6128217 30234115

[31] Saoudi González N , Salvà F , Ros J , et al. Unravelling the complexity of colorectal cancer: heterogeneity, clonal evolution, and clinical implications. Cancer. 2023;15:15. 10.3390/cancers15164020 PMC10452468 37627048

[32] Sarabia‐Sánchez MA , Robles‐Flores M . WNT signaling in stem cells: a look into the non‐canonical pathway. Stem Cell Rev Rep. 2024;20:52‐66. 37804416 10.1007/s12015-023-10610-5 PMC10799802

[33] van der Walt S , Schönberger JL , Nunez‐Iglesias J , et al. Scikit‐image: image processing in python. PeerJ. 2014;2:e453. 25024921 10.7717/peerj.453 PMC4081273

[34] Gouda MA , Subbiah V . Precision oncology for BRAF‐mutant cancers with BRAF and MEK inhibitors: from melanoma to tissue‐agnostic therapy. ESMO Open. 2023;8:100788. 36842301 10.1016/j.esmoop.2023.100788 PMC9984800

[35] Piercey O , Tie J , Hollande F , Wong H‐L , Mariadason J , Desai J . BRAFV600E‐mutant metastatic colorectal cancer: current evidence, future directions, and research priorities. Clin Colorectal Cancer. 2024;23:215‐229. 38816264 10.1016/j.clcc.2024.04.004

[36] Kasuga A , Nakagawa K , Nagashima F , et al. A phase I/Ib study of trametinib (GSK1120212) alone and in combination with gemcitabine in Japanese patients with advanced solid tumors. Invest New Drugs. 2015;33:1058‐1067. 26259955 10.1007/s10637-015-0270-2

[37] Virtanen P , Gommers R , Oliphant TE , et al. SciPy 1.0: fundamental algorithms for scientific computing in python. Nat Methods. 2020;17:261‐272. 32015543 10.1038/s41592-019-0686-2 PMC7056644

[38] Li Q , Geng S , Luo H , et al. Signaling pathways involved in colorectal cancer: pathogenesis and targeted therapy. Sig Transduct Target Ther. 2024;9:266. 10.1038/s41392-024-01953-7 PMC11456611 39370455

[39] Kabiri Z , Greicius G , Zaribafzadeh H , Hemmerich A , Counter CM , Virshup DM . Wnt signaling suppresses MAPK‐driven proliferation of intestinal stem cells. J Clin Invest. 2018;128:3806‐3812. 30059017 10.1172/JCI99325 PMC6118584

[40] Xu H , Hao Z , Wang Y , et al. Liquid tumor microenvironment enhances WNT signaling pathway of peritoneal metastasis of gastric cancer. Sci Rep. 2023;13:11125. 37429893 10.1038/s41598-023-38373-6 PMC10333202

[41] Fennell LJ , Kane A , Liu C , et al. APC mutation Marks an aggressive subtype of BRAF mutant colorectal cancers. Cancer. 2020;12:1171. 10.3390/cancers12051171 PMC7281581 32384699

[42] Braicu C , Buse M , Busuioc C , et al. A comprehensive review on MAPK: a promising therapeutic target in cancer. Cancer. 2019;11:1618. 10.3390/cancers11101618 PMC6827047 31652660

[43] Guo Y‐J , Pan W‐W , Liu S‐B , Shen Z‐F , Xu Y , Hu L‐L . ERK/MAPK signalling pathway and tumorigenesis. Exp Ther Med. 2020;19:1997‐2007. 32104259 10.3892/etm.2020.8454 PMC7027163

[44] Coller HA . The paradox of metabolism in quiescent stem cells. FEBS Lett. 2019;593:2817‐2839. 31531979 10.1002/1873-3468.13608 PMC7034665

[45] Zhan T , Ambrosi G , Wandmacher AM , et al. MEK inhibitors activate Wnt signalling and induce stem cell plasticity in colorectal cancer. Nat Commun. 2019;10:2197. 31097693 10.1038/s41467-019-09898-0 PMC6522484

[46] Michels BE , Mosa MH , Grebbin BM , et al. Human colon organoids reveal distinct physiologic and oncogenic Wnt responses. J Exp Med. 2019;216:704‐720. 30792186 10.1084/jem.20180823 PMC6400532

[47] White M , Mills ML , Millett LM , et al. MAPK‐driven epithelial cell plasticity drives colorectal cancer therapeutic resistance. Nature. 2025;650:748‐758. 41286180 10.1038/s41586-025-09916-w PMC12916511

[48] Barnett AM , Mullaney JA , McNabb WC , Roy NC . Culture media and format alter cellular composition and barrier integrity of porcine colonoid‐derived monolayers. Tissue Barriers. 2024;12:2222632. 37340938 10.1080/21688370.2023.2222632 PMC11042055

