# Supplementary material for: Impact of non‐genetic heterogeneity of BRAF‐mutant colon cancer organoids on growth kinetics, drug sensitivity and Wnt dynamics
Source: Int J Cancer. 2026 Mar 15;159(2):490–500. doi: 10.1002/ijc.70453 (PMC13193477; doi:10.1002/ijc.70453)
Supplement: Supplementary file 1 — FIGURE S1: Organoid growth and metabolic activity in dependence of seeding. FIGURE S2: Dose response to trametinib at different organoid seeding. FIGURE S3: Organoids cultured under growth factor‐supplemented conditions. FIGURE S4: Verification of size similarity between plated cystic and solid. [file IJC-159-490-s001.pdf]

Impact of Non-Genetic Heterogeneity of BRAF-mutant Colon Cancer Organoids on Growth Kinetics, Drug Sensitivity and Wnt Dynamics

Viktoria Zieger, Ellen Woehr, Jasmin Traichel, Tilman Brummer, Roland Zengerle, Sabrina Kartmann, Stefan Zimmermann

<u>Table of contents:</u>

Supplementary Figures

- **Supplementary Figure S1**: Organoid growth and metabolic activity in dependence of seeding density and organoid diameter.
- **Supplementary Figure S2**: Dose response to trametinib at different organoid seeding densities.
- **Supplementary Figure S3**: Organoids cultured under growth factor-supplemented conditions vs. organoids cultured under GF-depleted conditions.
- **Supplementary Figure S4**: Verification of size similarity between plated cystic and solid organoids prior to drug treatment for organoids under growth factor-depleted conditions (COCM).

Supplementary Tables (available in a separate Excel file)

- **Supplementary Table S1**: Organoid growth data
- **Supplementary Table S2**: CellTiter-Glo® Cell Viability data
- **Supplementary Table S3**: CellTiter-Glo® ATP standard curve data
- **Supplementary Table S4**: Wnt3a ELISA assay data

Supplementary Figures S1 - S4

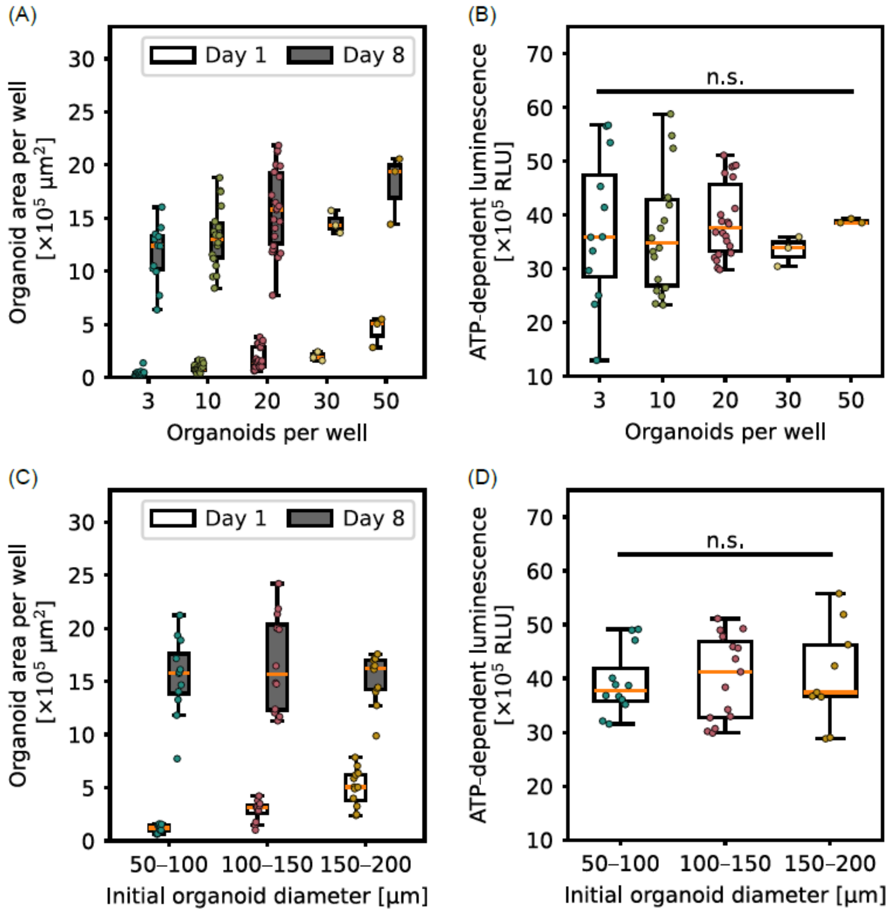

Supplementary Figure S1: (A) Total organoid area per well at days 1 and 8 plotted against organoid seeding density for organoids under growth factor-supplemented conditions (COCM+GF) (n=12,18,22,3,3). **(B)** Metabolic activity of untreated organoids measured on day 8 by the CellTiter-Glo 3D assay, which quantifies ATP levels per well (n=12,18,22,3,3; statistical analysis by Mann–Whitney test). **(C)** Total organoid area per well at days 1 and 8 plotted against initial organoid diameter (n=12 with 20 organoids per replicate). **(D)** ATP levels per well of untreated organoids on day 8 in relation to initial organoid diameter (n=12 with 20 organoids per replicate; statistical analysis by unpaired two-tailed t-test).

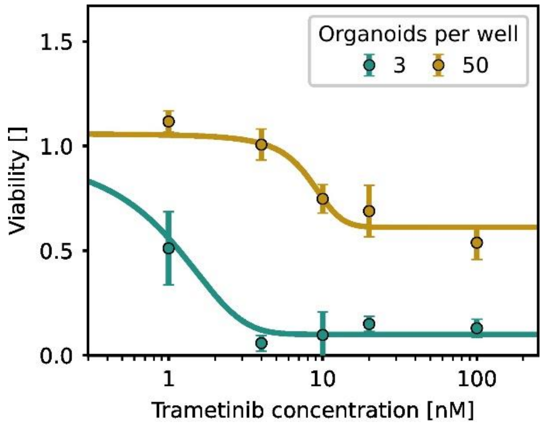

Supplementary Figure S2: Dose response to trametinib at different organoid seeding densities measured on day 8 post plating for organoids under growth factor-depleted conditions (COCM). Each condition: n≥3.

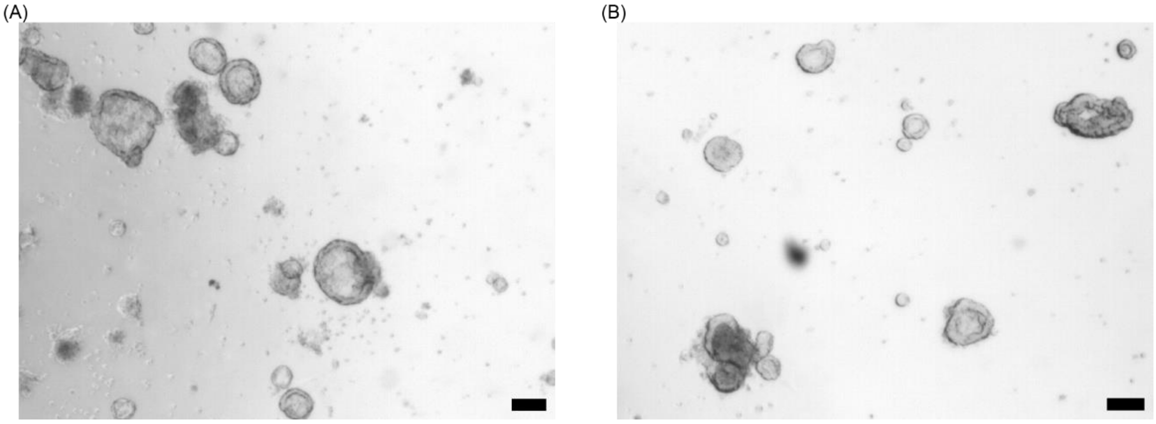

Supplementary Figure S3: Representative images of COL organoids harvested 3 days post-passaging, imaged within the reservoir of the PFD platform. **(A)** Organoids cultured under growth factor-supplemented conditions. **(B)** Organoids cultured under GF-depleted conditions. *Scale bar: 100 µm*.

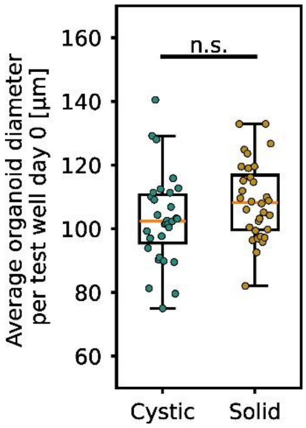

Supplementary Figure S4: Verification of size similarity between plated cystic and solid organoids prior to drug treatment for organoids under growth factor-depleted conditions (COCM). Measured total organoid area per well was divided by the number of organoids within well to receive average organoid diameter per well. (n≥31 with 20 organoids per replicate). Statistical analysis was performed using two-tailed unpaired t-tests. Differences with $p \geq 0.05$ were considered not significant (n.s.).
